# Intestinal Perforation Caused by Intestinal Tuberculosis of a Nepalese Man Living in a Low Tuberculosis‐Burden Country: A Case Report

**DOI:** 10.1002/ccr3.73521

**Published:** 2026-09-18

**Authors:** Shunya Matsushita, Kei Kanata, Shoma Matsushita, Yutaro Ito, Koshiro Ichijo, Masahiro Uehara

**Affiliations:** ^1^ Department of Respiratory Medicine Shimada Shiritsu Sogo Iryo Center Shizuoka Japan

**Keywords:** emergency surgery, intestinal tuberculosis, low tuberculosis‐burden country, perforation

## Abstract

Intestinal tuberculosis generally presents with chronic symptoms. However, in our case, the patient experienced rapid progression from intestinal obstruction to perforation. Even in low‐burden countries, intestinal tuberculosis should be considered when patients from high‐burden countries present with abdominal symptoms and characteristic computed tomography findings.

## 1 Introduction

Intestinal tuberculosis (TB) is a form of extrapulmonary TB and is classified within the broader category of gastrointestinal TB, which accounts for approximately 1%–3% of all TB cases [1].

Its symptoms are often nonspecific, making the disease difficult to diagnose. Additionally, the disease typically follows a chronic course, and acute onset is rare. Japan became a low‐burden TB country in 2021, and the number of TB cases has been declining [2, 3]. Thus, it is challenging to suspect intestinal TB when examining patients who complain of abdominal symptoms.

We experienced a rare case of intestinal perforation due to intestinal obstruction caused by TB in a Nepalese patient living in Japan.

## 2 Case History/Examination

The patient was a 41‐year‐old Nepalese man. He had been residing in Japan for 1.5 years. He had a history of smoking three cigarettes per day between the ages of 18 and 30, but had quit smoking thereafter. He did not consume alcohol. He had not taken any antibiotics prior to presentation. He had no history of diabetes mellitus, malignancy, autoimmune disease, or use of immunosuppressive medications. He had lost approximately 6 kg over the past year. However, his body mass index (BMI) remained elevated at 29.7 kg/m^2^, and he did not report any chronic gastrointestinal symptoms. In June of a particular year after dinner, he developed abdominal pain and nausea, followed by one episode of vomiting. The next morning, his symptoms did not improve, and he came to our hospital. There was no history of ingestion of raw food.

When he came to our hospital, he had a fever of 38.1°C. The abdomen was distended and soft, with tenderness in the lower abdomen, but no rebound tenderness was noted. Laboratory test results showed a white blood cell count of 8800/μL (neutrophil count of 7234/μL, lymphocyte count of 1118/μL), and a C‐reactive protein (CRP) level of 1.03 mg/dL. Both the human immunodeficiency virus (HIV) antibody test and serum β‐D‐glucan were negative.

**FIGURE 1:**
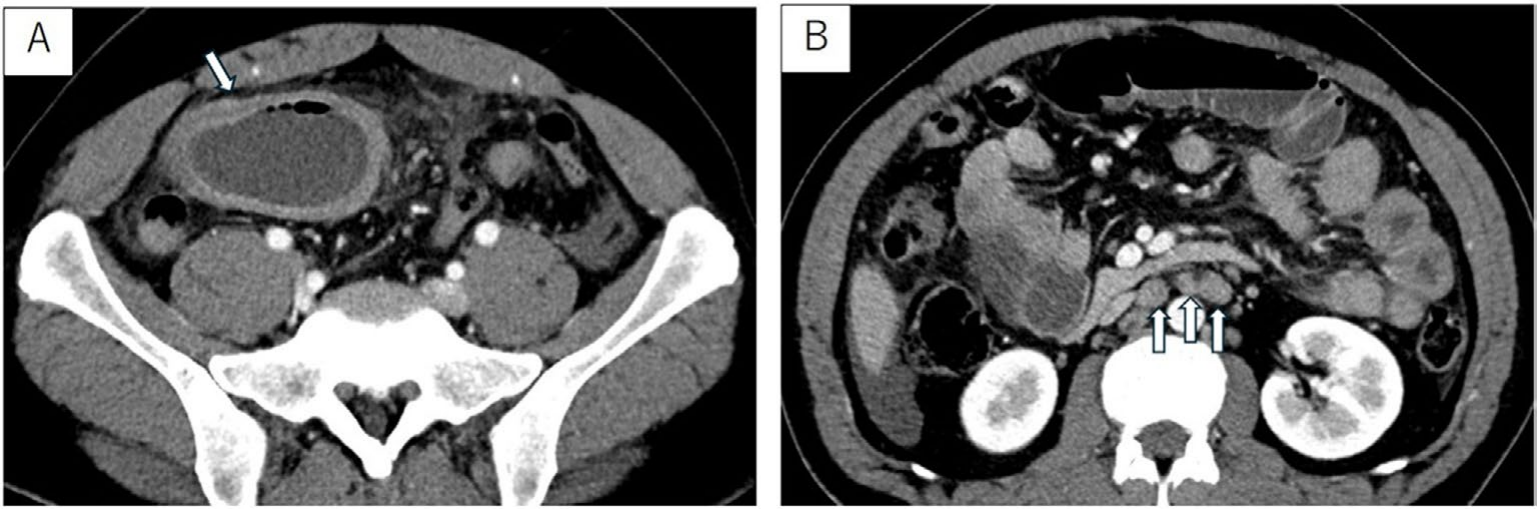
Contrast‐enhanced abdominal computed tomography (CT) performed at the time of admission revealed continuous dilatation of the small intestine, symmetric wall thickening, increased surrounding fat density, and enlarged lymph nodes (A). Multiple enlarged lymph nodes measuring more than 1 cm were also seen in the abdominal para‐aortic region (B). Arrows indicate the thickened small intestinal wall (A) and enlarged para‐aortic lymph nodes (B).

Contrast‐enhanced abdominal computed tomography (CT) showed continuous dilatation and symmetric wall thickening of the small intestine, accompanied by increased surrounding fat density and enlarged lymph nodes. Multiple enlarged lymph nodes measuring more than 1 cm were also seen in the abdominal para‐aortic region (Figure 1). Chest radiography and chest CT performed on admission showed no active pulmonary lesions. The patient was hospitalized and managed with fasting and rehydration. A gastric tube was placed, and antimicrobial therapy with cefmetazole was started. Cefmetazole was selected empirically in accordance with our hospital protocol for presumed intra‐abdominal infection in patients without a history of prior antibiotic use or recent hospitalization.

**FIGURE 2:**
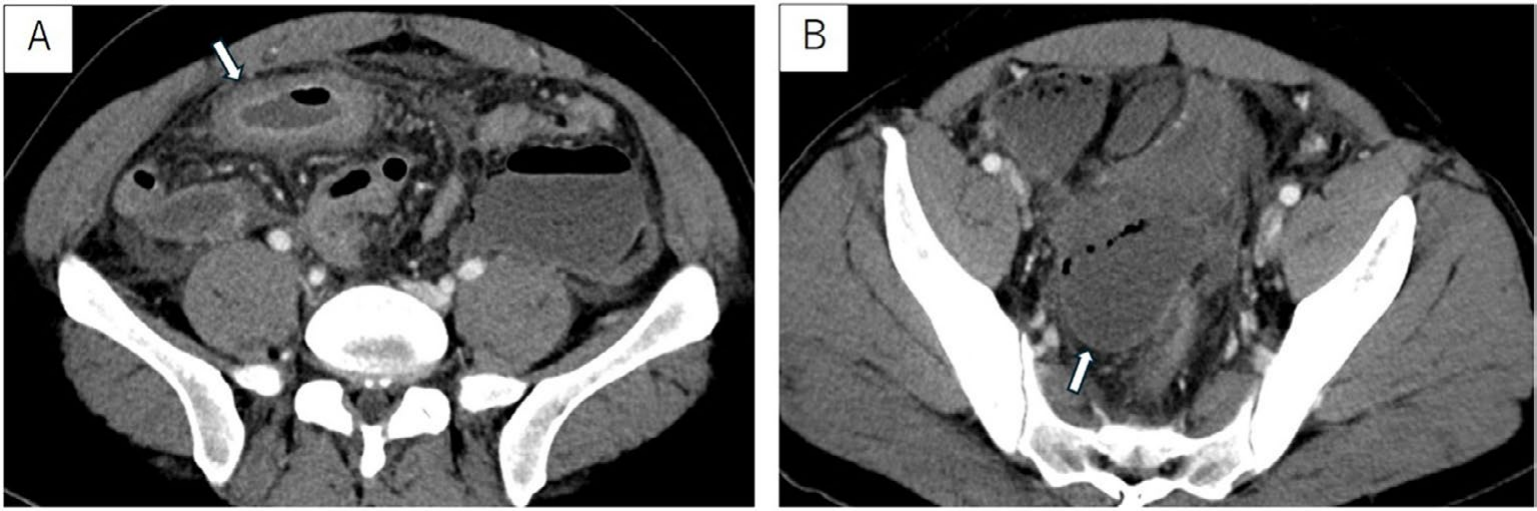
Contrast‐enhanced abdominal CT on the third day of admission showed free air in the abdominal cavity, increased fat density around the small intestine (A), and fluid accumulation in the abdominal cavity that indicated an intra‐abdominal abscess (B). Arrows indicate the affected small intestinal segment (A) and intra‐abdominal fluid collection (B).

On the second day of admission, the abdominal pain worsened and did not improve on the third day. Repeat laboratory tests showed a highly elevated CRP level of 32.86 mg/dL, and a procalcitonin level of 1.46 ng/mL. Contrast‐enhanced abdominal CT showed free air in the abdominal cavity, increased fat density around the small intestine, and fluid accumulation in the abdominal cavity that indicated an intra‐abdominal abscess (Figure 2). The diagnosis of small intestinal perforation was made, and emergency surgery was performed. A midline laparotomy revealed feculent peritonitis with fibrinous exudate, most prominent in the pelvis. A 2‐mm perforation and approximately 30 cm of edematous distal ileum were identified. The affected segment was resected, and a stapled side‐to‐side anastomosis was performed. After peritoneal irrigation with 20 L of saline, three drains were placed, and the abdomen was closed in layers.

## 3 Differential Diagnosis

Intestinal TB, Crohn's disease, and malignant tumors were considered as differential diagnoses. Although the lesion was in the ileocecal region, the absence of skip lesions made intestinal TB more likely than Crohn's disease. Malignant tumors are more common in the large intestine than in the small intestine. In this case, no mass lesion was identified on contrast‐enhanced CT images in both axial and coronal planes. However, we acknowledge that small‐bowel tumors can occasionally be missed on imaging. Thus, a definitive diagnosis could not be made until the final pathological results were available.

## 4 Conclusions and Results

**FIGURE 3:**
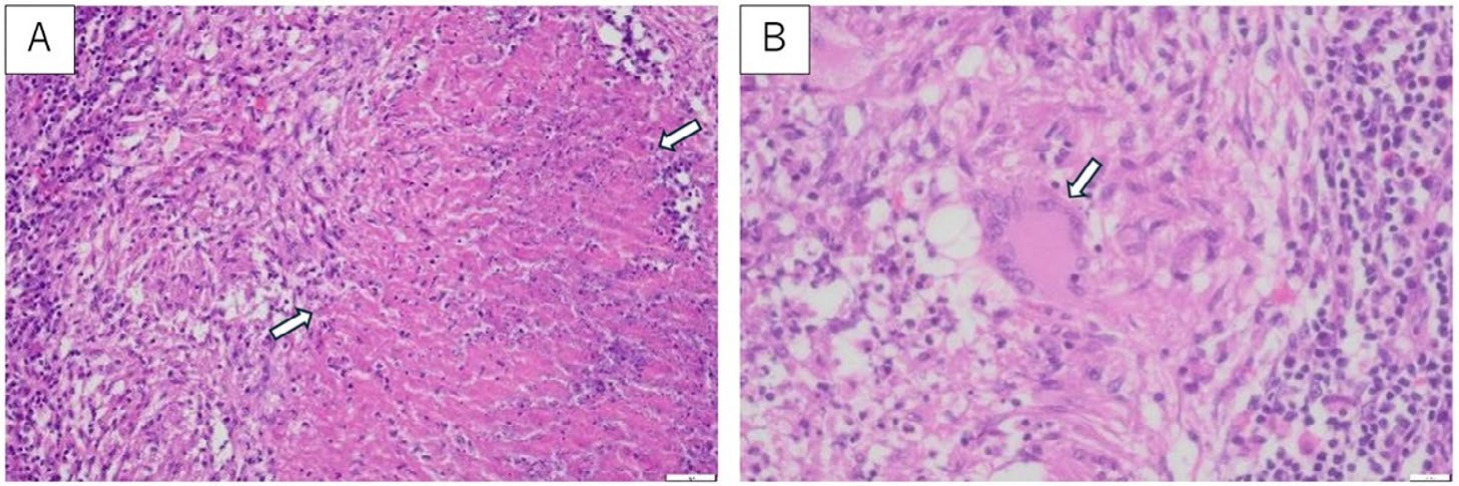
Histopathological examination of the resected small intestine showed epithelioid granulomas with caseous necrosis (A; H&E staining, ×200) and Langhans giant cells (B; H&E staining, ×400). Arrows indicate caseous necrosis (A) and a Langhans giant cell (B).

Histopathological examination of the resected small intestine showed epithelioid granulomas with caseous necrosis and Langhans giant cells (Figure 3). Additionally, the TB polymerase chain reaction (PCR) test of the ascitic fluid was positive. Thus, we diagnosed intestinal perforation caused by intestinal TB.

TB isolated from the ascitic fluid was sensitive to all major drugs, and anti‐TB drug therapy was promptly initiated. For the first 2 months, the patient was treated with a 4‐drug combination of isoniazid, rifampicin, ethambutol, and pyrazinamide. For the next 4 months, the patient was treated with a 2‐drug combination of isoniazid and rifampicin. During treatment, no side effects were observed; abdominal pain disappeared, and the CRP level normalized. Contrast‐enhanced abdominal CT, performed 3 months after the initiation of treatment, showed that the abscess had resolved and the enlarged lymph nodes had decreased in size. The patient completed 6 months of treatment. No recurrence was observed after that.

## 5 Discussion

Intestinal TB is a form of extrapulmonary TB and is classified within the broader category of gastrointestinal TB, which accounts for approximately 1%–3% of all TB cases [1]. The symptoms of intestinal TB are often nonspecific, such as abdominal pain, weight loss, fever, diarrhea, constipation, and bloody stools. Moreover, the clinical picture is often similar to that of Crohn's disease or malignant tumors, making differentiation problematic [4]. Japan became a low‐burden TB country in 2021, and the number of TB cases has been declining [2, 3]. Thus, it is challenging to suspect intestinal TB when examining patients who complain of abdominal symptoms.

CT findings are crucial for differentiation purposes. Intestinal TB is most commonly seen in the ileocecal region and is usually localized to a short segment. The presence of lymph nodes larger than 1 cm is more common in intestinal TB [5]. Crohn's disease is most commonly seen in the ileocecal area, and skip lesions are highly diagnostic. Asymmetric intestinal wall thickening is highly specific [6]. Malignant tumors are often seen in the large intestine, where the lesions are continuous and the intestinal wall shows a mass‐like contrast enhancement.

In this case, the findings included ileocecal involvement, short‐segment disease, enlarged lymph nodes measuring more than 1 cm, absence of skip lesions, and symmetric wall thickening. Furthermore, this patient was from Nepal, a high‐burden TB country. Thus, it is important to suspect intestinal TB even in low‐burden countries when patients from high‐burden countries present with abdominal symptoms and characteristic CT findings.

The median time from symptom onset to intestinal TB diagnosis has been reported to be 13 weeks [7], and intestinal TB generally follows a chronic course. Intestinal perforation occurs in 4%–7.6% of cases of intestinal TB, and the mortality rate is as high as 30% [8]. In this case, the patient presented with intestinal obstruction and developed intestinal perforation within a few days, and there are no previous reports describing a similar course.

Ammara et al. reported a case in which a patient diagnosed with intestinal TB was able to avoid perforation. He came to the hospital with intestinal obstruction and was diagnosed with abdominal TB after diagnostic surgery [9]. In this report, symptoms worsened relatively slowly; however, in our case, the patient visited our hospital due to a sudden deterioration of symptoms within 1 day, making an earlier diagnosis difficult. However, because the CT findings were typical and the patient had experienced weight loss over the past year, it is possible that perforation could have been prevented by early diagnostic surgery, and this serves as a warning to clinicians.

As reported, treatment generally proceeds smoothly if anti‐TB drug treatment is administered [10]. In this case, the patient quickly recovered after receiving anti‐TB drug treatment. Therefore, suspecting intestinal TB and making an early diagnosis are key to effective treatment.

The incidence of TB is declining in Japan, and the chances of encountering intestinal TB are also decreasing. In patients from high‐burden countries who present with abdominal symptoms, it is important to suspect intestinal TB. Even subtle signs such as weight loss should not be overlooked, and the possibility of acute perforation must be considered.

We experienced a rare case of intestinal TB that progressed from intestinal obstruction to perforation within a few days. Even in low‐burden countries, intestinal TB should be considered when patients from high‐burden countries present with abdominal symptoms and characteristic CT findings.

## Author Contributions

**Shunya Matsushita:** writing – original draft. **Kei Kanata:** supervision. **Shoma Matsushita:** supervision. **Yutaro Ito:** supervision. **Koshiro Ichijo:** supervision. **Masahiro Uehara:** supervision.

## Funding

The authors have nothing to report.

## Ethics Statement

Ethical approval was not required for this case report in accordance with institutional guidelines. The authors declare that appropriate written informed consent was obtained for the publication of this manuscript and accompanying images.

## Consent

Written informed consent from the patient was obtained for publication according to journal guidelines.

## Conflicts of Interest

The authors declare no conflicts of interest.

## Data Availability

The data that support the findings of this study are available from the corresponding author upon reasonable request.
